# Higher Revision Rates With In Situ Decompression as Compared to Ulnar Nerve Transposition for Cubital Tunnel Syndrome: A Meta-Regression Analysis

**DOI:** 10.7759/cureus.68116

**Published:** 2024-08-29

**Authors:** Rachel Reichenbach, Nicholas A Chartrand, Chad Stecher, Sean P Renfree, Michael Stickels, Joshua W Hustedt

**Affiliations:** 1 Department of Orthopaedic Surgery, University of Arizona College of Medicine - Phoenix, Phoenix, USA; 2 College of Health Solutions, Arizona State University, Phoenix, USA; 3 Department of Orthopaedic Surgery, University of Arizona College of Medicine - Tuscon, Tucson, USA

**Keywords:** in situ decompression, ulnar nerve transposition, cubital tunnel, cubital neuropathy, cubital tunnel surgery, systematic review and meta-analysis

## Abstract

The purpose of this study was to examine the effect of follow-up time on revision rates of in situ decompression and ulnar nerve transposition for the surgical treatment of cubital tunnel syndrome.

A comprehensive literature search was performed to identify prospective and retrospective primary comparison studies assessing the revision rates of in situ decompression and ulnar nerve transposition for the treatment of cubital tunnel syndrome. Meta-regression analyses were used to assess the effect of average study follow-up on the revision rates of both cubital tunnel syndrome treatments. Modeling results were then used to estimate revision rates between decompression and transposition at increasing follow-up times.

Sixteen studies including 2,225 patients were included. Average study follow-up time was a statistically significant moderator of revision rates. Model predictions show that in situ decompression operations had an increased risk for revision as compared to ulnar transposition after 48 months of follow-up. In studies with follow-up time ≥48 months, revision rates for in situ decompression (11.9%) were significantly greater than in ulnar transposition (3.2%).

In situ decompression for cubital tunnel syndrome is associated with an increased risk of revision surgery as compared to ulnar nerve transposition, particularly when assessed at longer follow-up intervals. The effect of follow-up duration on revision rates demonstrates the need for additional studies to compare outcomes of these operative approaches at follow-up times ≥48 months. This study provides evidence that ulnar nerve transposition may ultimately lead to lower revision rates and demonstrates the need for prospective, randomized trials to corroborate this effect.

## Introduction and background

Cubital tunnel syndrome is the second most common compressive neuropathy of the upper extremity [1-4]. Despite the high incidence of cubital tunnel syndrome, there is still no consensus regarding the optimal operative treatment between in situ decompression (decompressing but leaving the nerve in its normal anatomical location) and ulnar nerve transposition (decompressing and moving the nerve anterior to the medial epicondyle). For certain patients, such as those with post-traumatic cubital tunnel syndrome or with obvious pre-operative subluxation, transposition may be superior [1,2]. However, in cases of pure cubital tunnel syndrome, there is no clear gold standard technique. Studies comparing decompression with transposition have found differing results on surgical outcomes, especially with respect to revision. To date, only four randomized prospective trials have been conducted [5-8]. Three of these trials were limited to one year of postoperative follow-up [5,6,8]. The fourth trial had five years of follow-up but only enrolled 70 patients, with no revisions found in either group [7]. The overall conclusion of these trials is that there is no difference in revision rates between decompression and transposition. However, it may be that these trials were underpowered and/or lacking in follow-up time to show a difference if one were to exist. 

Recently published cohort studies have called into question the equivalence of decompression versus transposition with findings showing significantly higher revision rates with a decompression technique. Staples et al. found a rate of 13% in decompression versus 3% in transposition at a minimum of one year follow-up [3]. However, they qualified this finding by describing greater morbidity, narcotic consumption, and patient-reported disability associated with transposition. Hutchinson et al. similarly found a rate of 25% in decompression versus 12% in transposition at a minimum of five years follow-up [9]. In contrast, several other studies have concluded that in situ decompression is associated with fewer complications [5,6]. The differing conclusions of these studies suggest that further work needs to be done to examine the overall risk of revision with decompression versus transposition.

In addition, follow-up time in studies comparing decompression and transposition varies widely, from only a few months to more than 10 years. We observed an association between the length of study follow-up and revision rates, with longer follow-up time resulting in higher rates of revision with a decompression technique. Accordingly, the present study was designed to examine the effect of follow-up time on revision rates of in situ decompression and ulnar nerve transposition for the operative treatment of cubital tunnel syndrome. The hypothesis was that in the short term, both in situ decompression and ulnar nerve transposition result in similar outcomes, but in the long term, decompression results in an increased need for revision.

This article was previously presented as a meeting abstract at the American Association for Hand Surgery (AAHS) Annual Meeting on January 10, 2024.

## Review

Methods and materials

**Figure 1:**
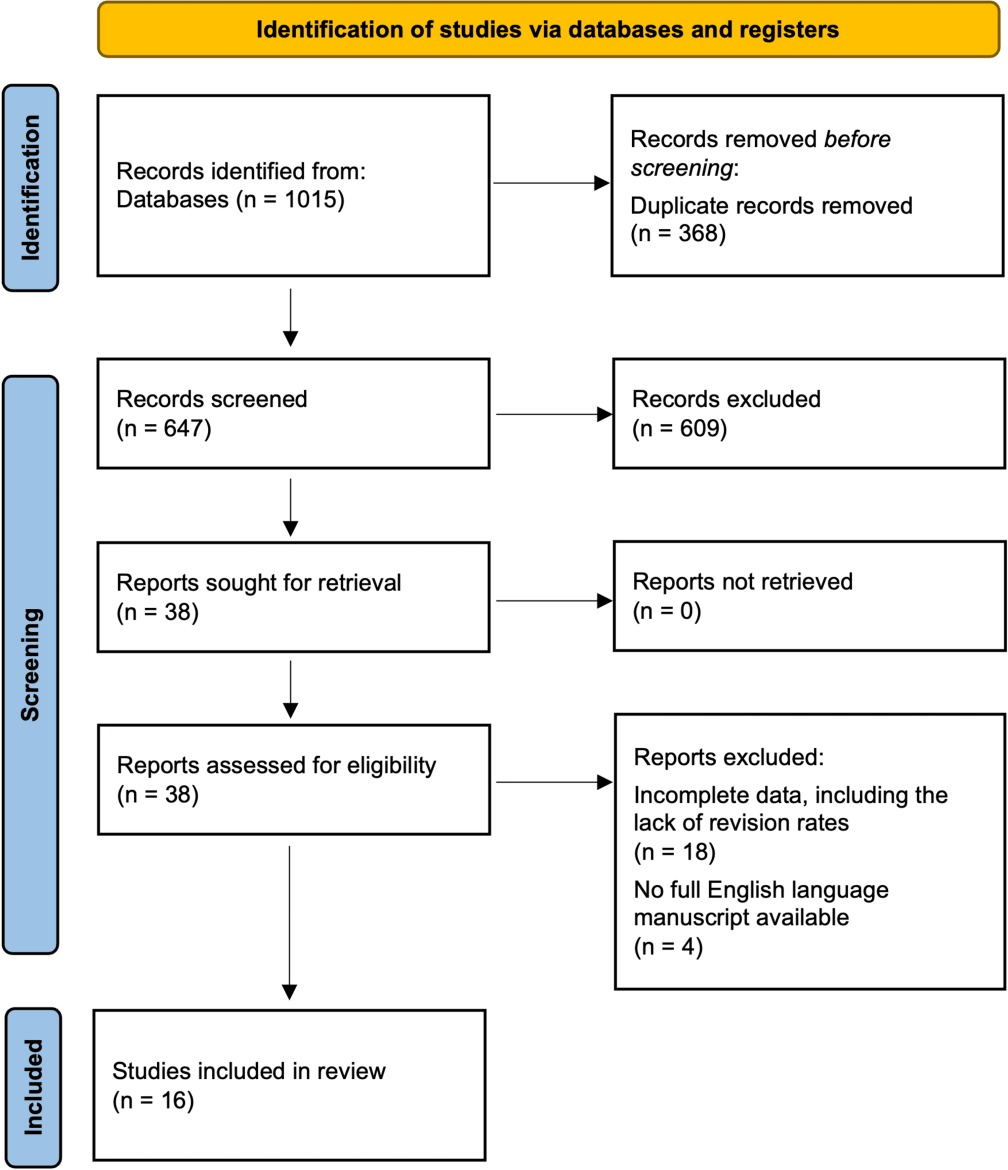
PRISMA flowchart. PRISMA: Preferred Reporting Items for Systematic Reviews and Meta-Analyses

This study was a systematic review and meta-analysis of all currently published studies comparing in situ decompression to ulnar nerve transposition in patients with cubital tunnel syndrome. A meta-regression was utilized to examine the effect of follow-up time on rates of revision surgery. The review was registered on the International Prospective Register of Systematic Reviews database 441563 and conducted in accordance with the Preferred Reporting Items for Systematic Reviews and Meta-Analyses (PRISMA) guidelines, as seen in Figure 1.

Study Selection and Outcome Measure

A comprehensive literature search was performed in PubMed, Embase, OVID, Medline, Google Scholar, and Cochrane Library to identify prospective and retrospective studies comparing the revision rates of in situ decompression and ulnar nerve transposition for the treatment of cubital tunnel syndrome. The following search terms were used: cubital tunnel syndrome, ulnar nerve entrapment, ulnar nerve compression, decompression, and transposition. Retrospective studies were included due to the relative paucity of randomized controlled trials comparing in situ decompression to ulnar nerve transposition for the treatment of cubital tunnel syndrome. Six hundred and forty-seven papers were initially identified across the databases. Study inclusion criteria were (1) idiopathic cubital tunnel syndrome and (2) primary comparison studies, including simple in situ decompression versus ulnar nerve transposition, with discrete data on revision rates for each procedure. In contrast, exclusion criteria included (1) incomplete data or outcomes, including the lack of revision rates, (2) inclusion of endoscopic cubital tunnel release or medial epicondylectomy in place of open simple decompression, and (3) no full English language manuscript available. Sixteen studies, with 2,225 patients, met these inclusion criteria [3,5-19]. The characteristics, demographics, and average follow-up time of each study are listed in Table 1. All studies were evaluated for quality and risk of bias via the appropriate standardized risk assessment tools, RoB-2 for the randomized controlled trials and ROBINS-I for the non-randomized studies, as demonstrated in Table 2andTable 3, respectively.

**Table 1:** Characteristics of studies included in the meta-analysis. Sample size (*n*) in each study is subdivided into in situ decompression and ulnar nerve transposition. RCTs are indicated under the study design. RCT: randomized controlled trial

Study	Year	Journal	Country of origin	Design	Total *n*	*n* (decompression)	*n* (transposition)	Mean age	Sex (% male)	Average follow-up (months)
Izadpanah et al. [10]	2021	American Association for Hand Surgery	USA	Retrospective	41	17	24	54	76	32
Hutchinson et al. [9]	2021	American Association for Hand Surgery	USA	Retrospective	132	73	59	48	37	60
Staples et al. [3]	2018	J Hand Surg Am	USA	Prospective	125	47	78	50	53	32
Zhang et al. [11]	2017	J Hand Surg Am	USA	Retrospective	247	157	90	51	43	3
Kamat et al. [12]	2014	Acta Neurochir	Iran	Retrospective	480	179	301	50	69	3
Bacle et al. [13]	2014	Ortho Traumatol Surg Res	France	Retrospective	306	44	262	54	57	92
Sousa et al. [14]	2014	Revista Brasileria Ortopedia	Portugal	Retrospective	97	64	33	51.9	57	10
Mitsionis et al. [15]	2010	J Shoulder Elbow Surg	Greece	Retrospective	73	34	39	51	68	37
Keiner et al. [8]	2009	Acta Neurochir	Germany	Prospective, RCT	33	17	16	49	67	63
Biggs and Curtis [6]	2006	Neurosurgery	Australia	Prospective, RCT	44	23	21	59	80	42
Bartels et al. [5]	2005	Neurosurgery	Netherlands	Prospective, RCT	152	75	77	47	62	12
Gervasio et al. [7]	2005	Neurosurgery	Italy	Prospective, RCT	70	35	35	53	68	47
Bimmler and Meyer [16]	1996	Annals of Hand and Upper Limb Surgery	Switzerland	Prospective	79	31	48	45	64	76
Foster and Edshage [17]	1981	J Hand Surg	USA/Switzerland	Prospective	48	29	19	52	56	50
Chan et al. [18]	1980	Neurosurgery	Canada	Retrospective	235	115	120	54	75	22
Paine [19]	1970	Canadian Journal of Surgery	Canada	Prospective	63	52	11	51	81	12

**Table 2:** Results of RoB-2 standardized risk assessment tool, risk of bias assessment for randomized controlled trials. Domains for RoB-2: D1: bias due to randomization; D2: bias due to deviations from intended intervention; D3: bias due to missing data; D4: bias due to outcome measurement; D5: bias due to selection of reported results Judgment key: x: high risk of bias; -: moderate risk of bias; +: low risk of bias

Study	D1	D2	D3	D4	D5	Overall
Keiner et al. [8]	-	+	+	-	+	-
Biggs and Curtis [6]	+	-	+	-	+	-
Bartels et al. [5]	+	+	+	-	+	-
Gervasio et al. [7]	+	+	+	+	+	+

**Table 3:** Risk of bias in non-randomized studies of interventions, ROBINS-I standardized risk assessment tool. Domains for ROBINS-I: D1: bias due to confounding; D2: bias due to selection of participants; D3: bias due to classification of interventions; D4: bias due to deviations from intended interventions; D5: bias due to missing data; D6: bias due to measurement of outcomes; D7: bias due to selection of the reported results Judgment key: x: high risk of bias; -: moderate risk of bias; +: low risk of bias

Study	D1	D2	D3	D4	D5	D6	D7	Overall
Izadpanah et al. [10]	-	+	+	+	-	-	+	-
Hutchinson et al. [9]	-	+	+	+	-	+	-	-
Staples et al. [3]	-	+	+	+	-	+	-	-
Zhang et al. [11]	-	+	+	+	-	+	+	-
Kamat et al. [12]	-	-	+	+	-	-	-	-
Bacle et al. [13]	-	+	+	+	-	-	+	-
Sousa et al. [14]	-	-	+	+	-	-	+	-
Mitsionis et al. [15]	-	-	+	+	-	-	+	-
Bimmler and Meyer [16]	-	-	+	+	-	-	+	-
Foster and Edshage [17]	-	-	+	+	-	-	+	-
Chan et al. [18]	-	-	+	+	-	-	+	-
Paine [19]	-	-	+	+	-	-	+	-

Statistical Analysis

**Figure 2:**
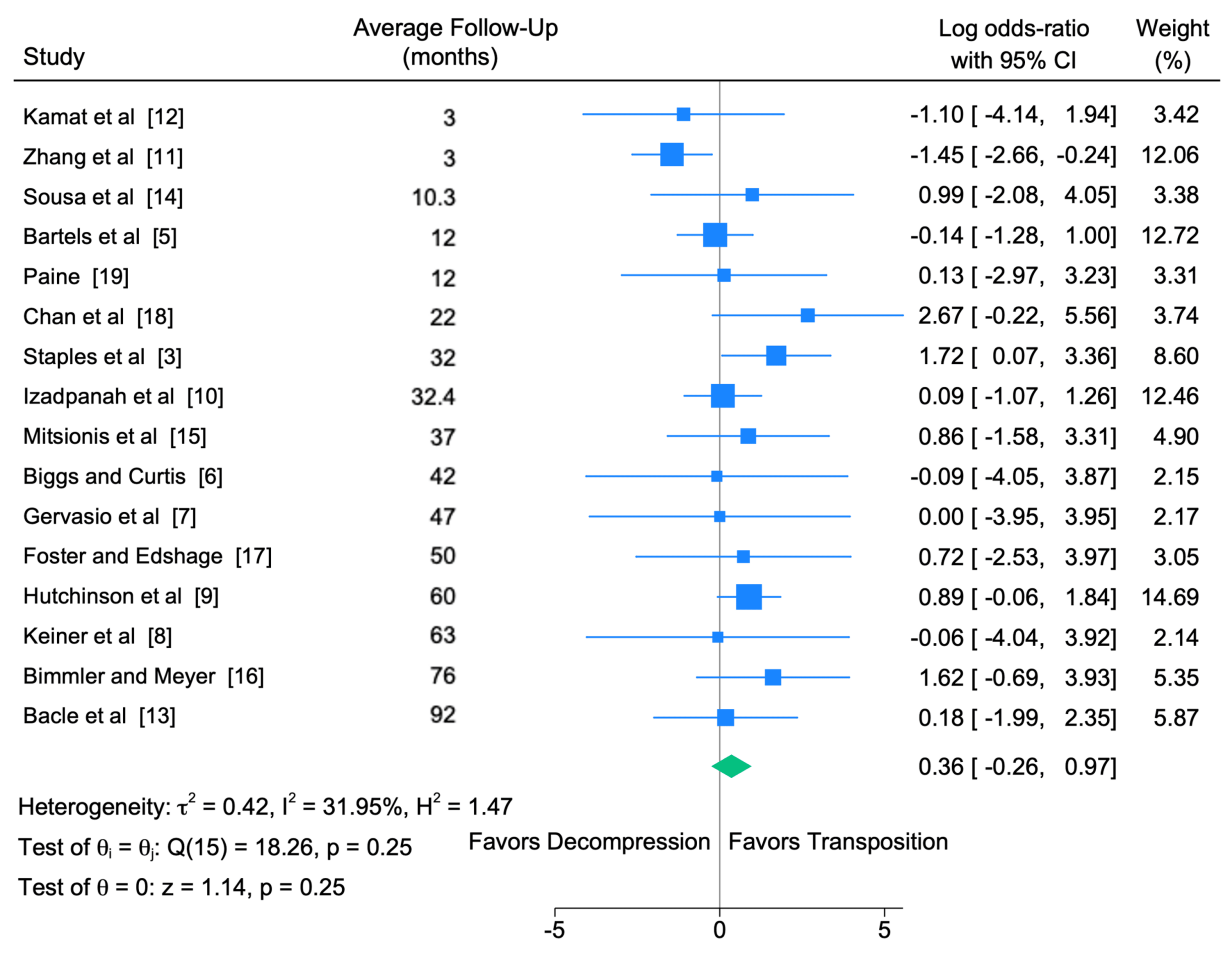
Forest plot representing the log odds ratio of each study included in the meta-analysis, sorted by average follow-up time.

We conducted a meta-analysis of revision rates across all studies included in our review (Table 4). The meta-analysis included a plot of heterogeneity of all studies, including the calculation of Cochran's Q test and the Higgins I² statistic, which are commonly used statistics to identify heterogeneous effect sizes [20,21]. The following thresholds for the interpretation of the I² statistic were used, 0-40%, 30-60%, 50-90%, or 75-100%, and were interpreted as not likely important, moderate, substantial, or considerable heterogeneity, respectively. We also calculated the mean follow-up time in each study. Figure 2 offers a forest plot representation of these results, by individual study. 

**Table 4:** Revision rates of patients who underwent in situ decompression vs. ulnar nerve transposition, by study.

Study	Revision rate (%): in situ decompression	Revision rate (%): ulnar nerve transposition
Izadpanah et al. [10]	7/17 (41)	9/24 (38)
Hutchinson et al. [9]	18/73 (25)	7/59 (12)
Staples et al. [3]	6/47 (13)	2/78 (3)
Zhang et al. [11]	4/157 (3)	10/90 (11)
Kamat et al. [12]	0/179 (0)	2/301 (1)
Bacle et al. [13]	1/44 (2)	5/262 (2)
Sousa et al. [14]	2/64 (3)	0/33 (0)
Mitsionis et al. [15]	2/34 (6)	1/39 (3)
Keiner et al. [8]	0/17 (0)	0/16 (0)
Biggs and Curtis [6]	0/23 (0)	0/21 (0)
Bartels et al. [5]	6/75 (8)	7/77 (9)
Gervasio et al. [7]	0/35 (0)	0/35 (0)
Bimmler and Meyer [16]	3/31 (10)	1/48 (2)
Foster and Edshage [17]	1/29 (3)	0/19 (0)
Chan et al. [18]	6/114 (5)	0/120 (0)
Paine [19]	2/52 (4)	0/11 (0)

**Figure 3:**
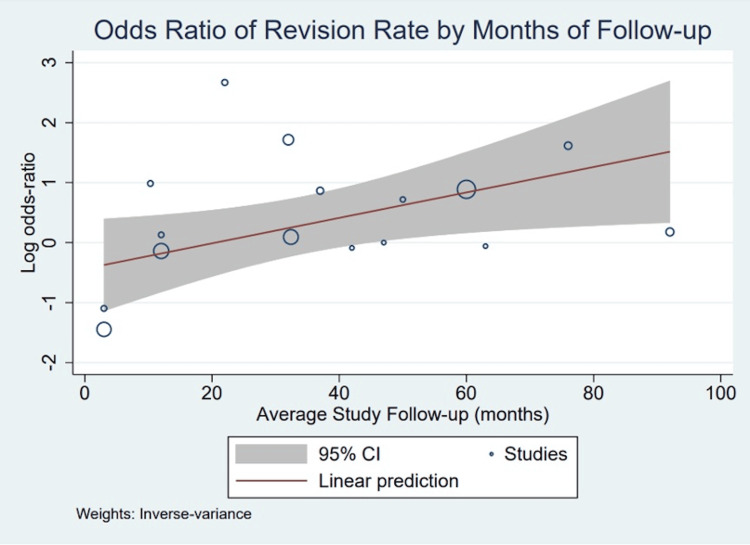
Bubble plot of revision rates by months of follow-up, with the estimated linear prediction and 95% confidence interval.

We then used meta-regression to examine the effect of follow-up time on revision rates between in situ decompression and ulnar nerve transposition. A bubble plot was used to graphically examine the estimated relationship between follow-up time and revision rate (Figure 3). The results of the meta-regression were then used to estimate the revision rates at differing follow-up times from one to 10 years. Effect sizes with numbers needed to harm were calculated for patients with greater than 48 months of follow-up. All statistical analyses were conducted in Stata/MP 16.1 using the "meta" commands. 

Results

Sixteen studies reporting on 2,225 patients were identified. Around 63% of the patients were male. The average study participant age across all studies was 51 years, with a range of 13-92 years. Studies were conducted in 11 separate countries. Follow-up times ranged from three to 92 months, with an average of 37 months.

There was a strong correlation between increasing follow-up time and the log odds ratio of revision rates for in situ decompression compared to ulnar transposition, as shown in Figure 3. Specifically, the estimated coefficient on average study follow-up time is 0.021 (95% CI: 0.002-0.040), which indicates that for one additional year of follow-up, the odds ratio of revision surgery for in situ decompression is 1.021. Based on these model estimates, we calculated the predicted odds ratio of revision. Model-adjusted average study follow-up estimates, along with odds ratio calculations and 95% confidence intervals, are shown in Table 5.

**Table 5:** Odds ratios of revision rate by years of follow-up based on model estimates, with 95% confidence interval values.

Average follow-up (years)	Odds ratio	95% CI
1	0.83	0.43-1.59
2	1.07	0.63-1.81
3	1.38	0.85-2.24
4	1.78	1.04-3.07
5	2.30	1.17-4.54
6	2.97	1.27-6.98
7	3.84	1.34-10.94
8	4.95	1.41-17.35
9	6.39	1.47-27.68
10	8.24	1.53-44.33

The results of the meta-regression suggest that there is a significantly increased risk of revision with in situ decompression after 48 months. As demonstrated in Table 6, when comparing average revision rates across all studies, in situ decompression yields a 5.8% revision rate, while ulnar nerve transposition yields a 3.5% revision rate. However, when assessing the average revision rate of studies with 48 months follow-up, the revision rates increase to 11.9% in the decompression group and decrease to 3.2% in the ulnar nerve transposition group. This higher rate of revision seen with decompression resulted in a number needed to harm of 11.5 after 48 months. The model-adjusted risk of revision at 10 years showed an eightfold risk of revision with decompression versus transposition (OR: 8.2; 95% CI: 1.5-44.3). 

**Table 6:** Meta-analysis of overall revision rates by follow-up time in patients who underwent in situ decompression vs. ulnar nerve transposition.

Operation	Overall revision rate	Revision rate <48 months	Revision rate ≥48 months
In situ decompression	5.8%	4.4%	11.9%
Ulnar nerve transposition	3.5%	3.6%	3.2%
			Number needed to harm (NNH): 11.5

Discussion

This study provides evidence that patients treated with an in situ decompression surgical technique may experience a higher need for revision as compared to ulnar nerve transposition and that this difference increases with increased follow-up time. The meta-regression model predicts an eightfold increase in the need for revision with decompression surgery at 10 years. Importantly, this analysis also suggests that the significance of this effect will not be apparent until after 48 months of follow-up time. Despite this finding, only 31% of current comparative studies include follow-up past 48 months.

Despite a high incidence of cubital tunnel disease, there is still a lack of data on outcomes between in situ and transposition surgery. In a 2019 meta-analysis comparing in situ decompression to ulnar nerve transposition, Said et al. found no difference in revision rates between in situ and transposition surgery [1]. However, they noted the large variability in study follow-up between two months and greater than five years. This observation led us to conduct the current study. 

The meta-regression data in this current study provides an important clarification on the role of follow-up time in determining the risks of operative treatment of cubital tunnel syndrome by in situ decompression. This study shows a clear, independent association of follow-up time and revision risk, with a difference in revision rates not becoming apparent until after 48 months. These results help clarify the differing results of prior studies, which either lacked sufficient follow-up or may have been underpowered to show a difference if one were to exist. This variation in follow-up time likely contributes to differential findings across meta-analyses examining in situ decompression and ulnar nerve transposition.

The strengths of this study are its inclusion of multiple studies conducted in varying locations. Our literature search resulted in a robust analysis of 2,225 patients in 11 different countries. Additionally, we chose to assess revision rates in operative treatments of cubital tunnel syndrome, a clinically important outcome and something that has only been assessed by one other meta-analysis to date [1].

There are several limitations of the data that are important to discuss. Despite the strong association between follow-up time and revision in this study, there was a large amount of variation in revision rates even among studies with longer follow-up times. We postulate that this variation is primarily due to the underpowering of a rare outcome (revision) in the setting of a paucity of large randomized controlled trials assessing these operative approaches. Secondarily, while indications for cubital tunnel revision are relatively clear, commonly based on patient-reported outcomes, and following the classic guidelines of persistent, recurrent, or new symptoms, originally described for carpal tunnel syndrome re-operation, now adopted for cubital tunnel syndrome [22,23], the complexity of decision-making for revision operations is both surgeon and patient specific. For instance, some surgeons may be hesitant to revise transposition, given the possible morbidity associated with further manipulation of the ulnar nerve, and therefore may be less inclined to offer revision operations to these patients. Third, although all included studies specifically assessed idiopathic cubital tunnel syndrome, we did not have sufficient data to assess factors such as co-existing chronic diseases or daily activity requirements, which may also play a role in revision rates in these patients. Future studies may choose to examine these factors in addition to revision rates.

Additionally, there is minimal consistency across studies when describing the specific type of ulnar nerve transposition technique chosen. Some studies subclassified the procedure into submuscular, intramuscular, and subcutaneous, while others did not. We reported revision rates for each of these approaches under ulnar nerve transposition, without distinguishing, as there was wide under-reporting of different subtypes across studies. Even for simple decompression, technical variations exist between surgeons. While this inconsistency reflects the broader lack of clarity as to which surgical approach to cubital tunnel syndrome treatment is superior, we acknowledge that a specific operative approach to transposition or decompression may have a differential effect on outcomes.

Furthermore, not all studies assessed were randomized controlled trials. Of the 16 studies assessed, only eight were prospective. Of those that were prospective, four were randomized controlled trials, and only one was conducted less than 15 years ago. While the relative deficit of current, randomized controlled trials reflects the broader lack of agreement upon operative approaches to cubital tunnel syndrome, this did limit the strength of the evidence presented in this meta-analysis study. While previous meta-analyses have been able to assess a greater number of randomized controlled trials, the additional inclusion criteria of discrete outcome data in this study, including revision rates, limited the pool of studies [1]. However, we aimed to address this limitation via our standardized risk assessment, which demonstrated that even the non-randomized studies were at low to moderate risk of bias across domains.

Despite these limitations, this study encompasses all currently available data to suggest that there is a consistent relationship between follow-up time and revision rates: a reality that must be considered when comparing long-term outcomes for patients undergoing in situ decompression or ulnar nerve transposition for cubital tunnel syndrome. This paper indicates both the necessity of conducting further randomized controlled trials assessing revision rates between these two operative approaches and the need to standardize an appropriately long follow-up timeline. The latter will be critical in establishing a larger body of research with accurate patient outcomes, which ultimately can guide surgeons in their choice of operative approaches to treating cubital tunnel syndrome.

## Conclusions

This meta-regression examined all available prospective and retrospective studies reporting revision rates following in situ decompression versus anterior transposition for cubital tunnel syndrome. There was a significantly higher revision rate in studies that had at least a 48-month follow-up time frame. However, very few studies have been conducted with long-term follow-up. Taking all studies into account, our statistical model suggests an eightfold increased revision risk with in situ decompression compared to anterior transposition at 10 years. These data suggest that there are higher rates of revision after in situ decompression versus anterior transposition. However, there may be many reasons for these findings, including the lack of well-designed, randomized, long-term outcome studies. We believe that a randomized study needs to be conducted with greater than 48 months follow-up to better determine the actual revision risk following in situ versus anterior transposition for cubital tunnel syndrome. 
